# Combined Multiomics Analysis Reveals the Role of *ANXA1* Methylation and miRNA‐Targeted *LDLR* in Polycystic Ovary Syndrome

**DOI:** 10.1002/rmb2.12664

**Published:** 2025-06-23

**Authors:** Yicen Ding, Lishi Huang, Mengju He, Fei Zhang, Yuanjun Jiang, Yani Kang

**Affiliations:** ^1^ School of Biomedical Engineering, Bio‐ID Center Shanghai Jiao Tong University Shanghai China; ^2^ Peace Maternity and Child Health Hospital School of Medicine, Shanghai Jiao Tong University Shanghai China

**Keywords:** epigenomics, metabolomics, multiomics, polycystic ovary syndrome, transcriptomics

## Abstract

**Purpose:**

Polycystic ovary syndrome (PCOS) is an endocrine syndrome that afflicts women of childbearing age, whose specific pathogenesis is unknown. Combined multiomics analysis on it is still lacking. The purpose of this study was to use combined multiomics analyses to learn about the development of PCOS.

**Methods:**

We randomly selected three PCOS mouse models and two control mice as the mouse group, as well as three PCOS patients and two normal women as the human group. The data were analyzed by multiomics analysis including methylomes, transcriptomics, and metabolomics. We explored the key genes involved in the occurrence and development of PCOS that were common in multiomics. Methylation‐specific polymerase chain reaction (MSP) and real‐time PCR (qPCR) experiments were performed to verify the reliability of the results.

**Results:**

The gene *ANXA1* was hypomethylated and highly expressed in both mouse and human samples. Meanwhile, *LDLR* had a lower expression in both mouse and human samples, targeted by an upregulated microRNA (miRNA) called has‐miR‐106a‐5p, which may relate to hyperandrogenemia.

**Conclusions:**

Epigenetic mechanisms have an impact on the development of PCOS. Both *ANXA1* and *LDLR* play important roles in the pathological process of PCOS and have the potential to be diagnostic markers and therapeutic targets.

## Graphical abstract

This study combined epigenomics, transcriptomics, and metabolomics to search for genes that play a role in the development of PCOS in PNA mouse models and PCOS patients. Two key genes, ANXA1 and LDLR, were finally identified, which may be involved in the pathological process of PCOS.

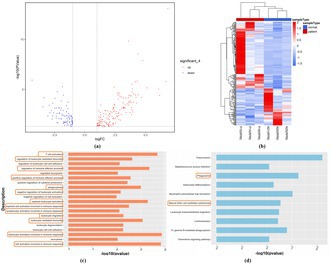

## 1 Introduction

Polycystic ovary syndrome (PCOS) is a common hormonal imbalance syndrome in women of reproductive age [1]. It is estimated that 8%–13% of women of childbearing age are affected by PCOS, but up to 70% of them remain undiagnosed [2]. Polycystic ovaries, anovulation, and hyperandrogenemia are the three main features of PCOS [2]. Studies have shown that patients with PCOS have impaired endometrial function and some degree of weakened oocyte capacity compared to healthy women [3, 4]. This is closely related to infertility (regardless of ovulatory status) and pregnancy complications in PCOS patients. In addition, PCOS can lead to insulin resistance (IR), menstrual disorders, hirsutism, obesity, acne, and oily skin. PCOS also comes with a host of complications such as endometrial cancer, type 2 diabetes, and cardiovascular disease [5, 6].

Due to the extreme heterogeneity of PCOS, there is currently no definitive diagnostic method. The Rotterdam criteria recommended by the Endocrine Society are generally used for diagnosis, which require at least two of the following three manifestations to appear: hyperandrogenism, ovulatory dysfunction, and polycystic ovaries [7]. PCOS cannot be cured, but its symptoms can be relieved with treatment [2]. The clinical treatment of PCOS is mainly drug therapy such as using clomiphene, letrozole, metformin, statins, or gonadotropins [8]. Surgeries can be used for patients who have resistance or severe side effects of drug treatments. For instance, a minimally invasive surgery, transvaginal hydrolaparoscopy (THL), provides an accurate image of the female pelvis. Thus, surgeries can be performed in the patient's ovarian capsule drilling [9].

The exact cause of PCOS has not been identified, but women with a family history of type 2 diabetes or obesity are at higher risk [10, 11]. Recent research advances in genetics and epigenetics have improved the understanding of the etiology and pathophysiology of PCOS, and certain loci associated with PCOS have stimulated the search for new therapeutic strategies [12]. Several studies have confirmed changes in the levels of histone acetylation, non‐coding RNA (ncRNA), and DNA methylation in PCOS [13]. RNA‐mediated DNA methylation (especially in promoter regions) leads to gene silencing at the transcriptional level. In active genes, promoters are usually hypomethylated [14]. A methylation histology study showed that PCOS patients have significant methylation level changes in several genes involved in a variety of physiological functions [15]. Abnormal histone modifications are associated with insulin‐related pathway dysfunction and may contribute to the development of PCOS [16]. Large amounts of aberrant long‐stranded ncRNAs were detected in various rodent models of PCOS and samples from different tissues of women with PCOS (serum, granulosa cells, follicular fluid, and ovarian cumulus cells) [17].

Also, PCOS is strongly associated with metabolomics. Abnormalities in pathways associated with steroidogenesis are of importance in the development of PCOS [18]. PCOS is usually characterized by increased levels of luteinizing hormone (LH) and gonadotropin‐releasing hormone (GnRH), resulting in ovulatory dysfunction and hyperandrogenemia [19]. Elevated androgen levels also impair the metabolic function of the pancreas, leading to IR symptoms [20]. Although the study of various omics has made great progress in the pathological process of PCOS, the combined multiomics analysis is still lacking. In this study, we conducted methyl‐CpG binding domain‐based capture and sequencing (MBD‐seq) and miRNA sequencing (miRNA‐seq) data from ovarian tissues of PNA mouse models and ovarian granulosa cells (GCs) of patients with PCOS to screen differentially expressed genes and differentially promoter‐methylated genes in PCOS. These data were jointly analyzed with metabolomics analysis and transcriptome sequencing (RNA‐seq) data to find differentially expressed miRNAs and differentially methylated genes in PCOS. Finally, through functional analysis, we aimed to identify and screen the biomarkers related to the diagnosis of PCOS and studied their biological functions, which are widely involved in metabolic functions, hormone synthesis, and immune‐inflammation in PCOS.

## 2 Materials and Method

### 2.1 Construction of PNA Mouse Model

Female mice were randomly mated with males. The discovery of the copulatory plug was identified as the first day of pregnancy in the female mice. After 16–18 days of gestation, the mice were randomly divided into two groups: The pregnant group was subcutaneously injected with 70 μL containing 350 μg dihydrotestosterone (DHT) and the control group with isopycnic sesame oil every day. The offspring of the DHT‐induced group were PNA mice, while the offspring of the control group were control mice. The mice were anesthetized and euthanized after 8 weeks, and the ovarian tissues were collected.

### 2.2 Participants' Information and Clinical Measurement

All of the participants (17 PCOS patients and 17 normal subjects) were recruited in Yuncheng Central Hospital of Shaanxi Province. Participants, between the ages of 20 and 35 from the same geographical region, agreed to participate in the study and provided written informed consent. The diagnostic criteria for PCOS patients are at least two Rotterdam ESHRE/ASRM (2003) criteria [21]. The control group were normal women of childbearing age and had normal menstrual cycles, no clinical symptoms related to PCOS, and no endometriosis or other chronic diseases, etc.

Clinical data were collected about 4 days after menstruation, including the number of follicles, preoperative serum hormone levels, and BMI. Participants' fasting whole blood was collected for 10 h. The Beckman UniCel DxI800 fully automatic chemiluminescence analyzer (Beckman Coulter, CA, USA) measures prolactin (PRL), LH, testosterone (T), thyroid stimulating hormone (TSH), and other hormone levels.

### 2.3 Isolation of GCs and Follicular Fluid Lipid

Participants were injected subcutaneously with 5000 IU of gonadotropin‐releasing hormone for 15 days, 7 days after ovulation, and follicle size was measured with ultrasound. When more than three follicles were detected with a diameter of 16 mm, participants were then injected with 6000 IU of human chorionic gonadotropin. There was no ovarian stimulating injection such as recombinant FSH or human menopausal gonadotropin. After 1.5 days, the oocytes were collected under ultrasound guidance. Then, oocytes were removed, and follicular fluid and GCs were collected by centrifugation. The collected GCs were washed twice with Dulbecco's modified Eagle medium. The follicular fluid lipid and GCs were quickly transferred to −80°C for preservation.

### 2.4 Extraction of Nucleic Acid

Mouse ovarian tissue samples and human GC samples were extracted by TRIzol (Invitrogen, Waltham, MA, USA) as recommended by the kit manufacturer's protocol, and the quality of RNA was assessed by 2% agarose denatured gel electrophoresis. Genomic DNA was extracted from mouse ovarian tissue samples and human GC samples using the QIAamp DNA Mini‐Kit (Qiagen, Hilden, Germany) according to the manufacturer's instructions, which use NanoDrop One (Thermo Scientific, Wilmington, USA) to identify the quality of genomic DNA.

### 2.5 MBD Sequencing and Data Analysis

The sequencing data for MBD‐seq were downloaded from our group's previous studies (https://www.ncbi.nlm.nih.gov/geo/; Accession Number: GSE156961 & GSE138575) [22, 23]. After MBD‐seq sequencing, trim_galore (version 0.4.3) was used to trim and filter the raw data to obtain clean reads. FastQC (version 0.11.5) was then used for quality inspection. Clean reads were then aligned to the mouse reference genome mm10 or the human reference genome hg38 using bowtie2 (version 2.1.0). Then, we use samtools(version 1.5) to filter and sort the data with MAPQ30 as the standard, and finally use picard (version 2.25.4) to deduplicate and get the .*bam* file. Differential analysis of methylated regions across the genome was performed using the edgeR (version 3.38.4) package with thresholds of |log_2_FoldChange| > 1 and *p* < 0.05. Subsequently, the distribution of genome‐wide differential methylation signals on chromosomes was visualized, and hierarchical cluster analysis of DMRs was performed to explore the differences in DNA methylation patterns between the two groups. Finally, the distribution of DMRs in genomic characteristic regions was analyzed by gene annotation, and DMGs were screened. GO enrichment analysis and KEGG pathway analysis were performed on the screened DMGs to explore the related biological pathways and functions of them in the occurrence and development of PCOS.

### 2.6 miRNA Sequencing and Data Analysis

The miRNA sequencing data used in this study for mouse ovarian and human GCs were downloaded from our previous studies (https://www.ncbi.nlm.nih.gov/geo/; Accession Number: GSE168166 & GSE138575) [23, 24]. Cutadapt (version 1.18) was used to trim and filter the raw miRNA‐seq data to obtain clean reads. This was followed by FastQC (version 0.11.5). The clean reads were then aligned to mouse or human mature miRNAs in miRBase (Release 22.1) using bowtie2 (version 2.1.0). Finally, samtools (version 1.5) was used to count and obtain the expression matrix of the sample miRNAs. Subsequently, the miRNA differential analysis was performed by edgeR (version 3.38.4) and DESeq2 (version 1.36.0) methods, and the threshold was set as |log_2_FoldChange| > 1 and *p* < 0.05, and the results of the two methods were intersected to obtain DemiRs. Then, the final DEmiRs were visualized by using the volcano map. Finally, hierarchical clustering analysis was performed on DEmiRs to explore the differences in miRNA expression patterns between the two groups, and a hierarchical clustering heat map was drawn. The potential target genes corresponding to the DEmiRs were predicted using the miRTarBase (Release 9.0) database. Subsequently, GO enrichment analysis and KEGG pathway analysis were performed to explore the relevant biological pathways and functions of the target genes in the occurrence and development of PCOS.

### 2.7 Transcriptome Sequencing and Data Analysis

The transcriptome sequencing data for mouse ovarian tissue and human GCs were derived from our previous studies (https://www.ncbi.nlm.nih.gov/geo/; Accession Number: GSE156961 & GSE138575) [22, 23]. After RNA‐seq sequencing, the raw offline data were trimmed and filtered using trim_galore (version 0.4.3) to obtain clean reads. FastQC (version 0.11.5) was then used for quality inspection. The clean reads were then aligned to the mouse reference genome mm10 or the human reference genome hg38 using HISAT2 (version 2.2.1). Finally, featureCounts (version 2.0.3) were used to count each gene according to the Ensembl gene‐level annotation to obtain the mRNA expression matrix. This is followed by subsequent data processing and analysis in R. The difference analysis was carried out by using edgeR and DESeq2 packages, and the threshold was set as |log_2_FoldChange| > 1 and *p* < 0.05. The analysis results of the two packages were intersected to obtain DEGs, and the final DEGs were visualized by using the volcano map. Finally, hierarchical clustering analysis was performed on DEGs to explore the differences in gene expression patterns between the two groups, and a heat map of sub‐clustering was drawn for visualization. The function of genes was enriched by the GO database from function and biological pathways involved. Pathway enrichment analysis was performed using the KEGG to explore the biological processes and pathways related to PCOS.

### 2.8 Validation of Promoter Methylation Level by MSP

Genomic DNA extracted from clinical samples was converted by bisulfite using the EZ DNA Methylation‐Gold Kit. In the presence of bisulfite, methylated cytosine does not undergo conversion, while unmethylated cytosine is converted to thymine. Primers designed for methylated and unmethylated promoter DNA sequences were used for PCR amplification. The primers used in MSP are shown in Table S1. The MSP result is shown in Figure S1.

### 2.9 Validation of Gene Expression Level by RT‐qPCR

The gene expression level of *ANXA1* was detected by RT‐qPCR. 500 ng total RNA was reverse‐transcribed into cDNA using PrimeScript RT Master Mix (RR037; TAKARA, Japan). The obtained cDNA was quantitatively detected by The Luna Universal qPCR Mix (M3003S; NEB, USA) in QuantStudio 3 (Applied Biosystems) according to the manufacturer's protocol. The primers used in RT‐qPCR are shown in Table S2.

### 2.10 Follicular Fluid Lipid Metabolites Analysis

Follicular fluid stored was restored at −80°C to room temperature and transferred 50 μL of follicular fluid into a 1.5 mL centrifuge tube. 200 μL of methanol precooled at 4°C, 400 μL of chloroform, and 170 μL of distilled waterwere thoroughly added and vortexed. Next, we centrifuged at a low temperature of 12 000 rpm at 4°C for 10 min to collect the lower clear liquid. Then, the lower clear liquid was transferred to a vacuum drying concentrator for low‐temperature freeze‐drying. The freeze‐dried sample was dissolved in 100 μL L (methylene chloride:isopropyl alcohol:methanol = 2:1:1 ([vol:vol:vol])), vortex thoroughly and centrifuged at 12 000 rpm for 10 min. 50 μL of supernatant was taken for UPLC‐MS mass spectrometry. The mass spectrometry data were compared with the Human Metabolome Database. Orthogonal partial least squares discriminant analysis (OPLS‐DA) modeling analysis was performed to determine the difference between the samples. The variable importance in projection (VIP) of lipid metabolites in follicular fluid was greater than 1, |log_2_ (FC)| ≥ 1, and *p*‐value < 0.05 were identified as differential metabolites.

### 2.11 Statistical Analysis

The software used for statistical analysis of data in this study is Statistical Package for Social Sciences (SPSS) (version 24.0). The difference between the two groups was determined by the two‐sided Student's *t*‐test; *p*‐value < 0.05 was considered a statistically significant difference.

## 3 Results

### 3.1 Sequencing Data Analysis of Prenatally Androgenized (PNA) Mouse and PCOS Patients

#### 3.1.1 Characterization of the PNA Mouse Model and PCOS Clinical Patients

Studies have shown that compared to other PCOS animal models, the mRNA levels of PNA mice are closest to those of PCOS patients [25]. Therefore, PNA mice were selected as PCOS animal samples in this study.

To confirm the establishment of the PNA mouse model, we randomly selected six PNA mice and six control mice and measured their estrus time, serum testosterone levels, and number of secondary follicles at 8 weeks of age (Table 1), which in the PNA mouse group were significantly higher than those in the control group. It is evident that we have successfully constructed the PNA mouse model.

**TABLE 1:** Comparison of characteristics of PNA group and control group.

Model feature	PNA (*n* = 6)	Control (*n* = 6)	*p*
duration of oestrus (%)	35 ± 5	18 ± 7	0.0014
testosterone level (nmol/L)	226.89 ± 13.90	187.45 ± 9.44	0.0002
number of secondary follicles	687 ± 145	402 ± 74	0.0016

Abbreviation: PNA, prenatally androgenized.

A total of 17 patients with PCOS and 17 control women of normal childbearing potential were candidates for ovarian GC samples. Patients with PCOS were selected according to the Rotterdam criteria [7]. Table 2 lists the clinical data for all participants. Comparing the clinical data of the PCOS patient group with the control group, the body mass index (BMI) index and LH levels of PCOS patients were significantly higher than those of the control group (*p* < 0.05), and there was no significant difference in other characteristics.

**TABLE 2:** Comparison of characteristics of PCOS Clinical Patients and controls.

Clinical feature	PCOS (*n* = 17)	Control (*n* = 17)	*p*
Age	29.3 ± 3.89	31.07 ± 4.79	0.293
BMI (kg/m^2^)	24.82 ± 3.36	21.95 ± 2.58	0.005
Waistline (cm)	75.2 ± 5.59	76.8 ± 6.27	0.596
Hipline (cm)	92 ± 4.92	91.53 ± 5.26	0.166
FBS (mmol/L)	5.1 ± 0.31	5.11 ± 0.35	0.737
FSH (mIU/mL)	5.01 ± 1.68	5.28 ± 2.13	0.742
E2 (pg/mL)	55.86 ± 44.08	55.67 ± 32.64	0.404
P (ng/mL)	0.78 ± 0.38	0.62 ± 0.2	0.184
PRL (ng/mL)	11.06 ± 6.62	11.31 ± 6.04	0.408
LH (mIU/mL)	7.76 ± 3.88	3.54 ± 2.25	0.004
T (ng/mL)	1.69 ± 0.94	1.54 ± 0.46	0.659
β‐HCG (mIU/mL)	0.98 ± 0.55	0.82 ± 0.62	0.217
Number of follicles ≥ 14 mm	13.1 ± 4.53	9.73 ± 2.94	0.033

Abbreviations: β‐HCG, beta‐human chorionic gonadotropin; BMI, body mass index; FBS, fasting blood sugar; E2, estrogen; LH, luteinizing hormone; P, progesterone; PRL, prolactin; T, testosterone.

#### 3.1.2 Analysis of Differentially Methylated Patterns

**FIGURE 1:**
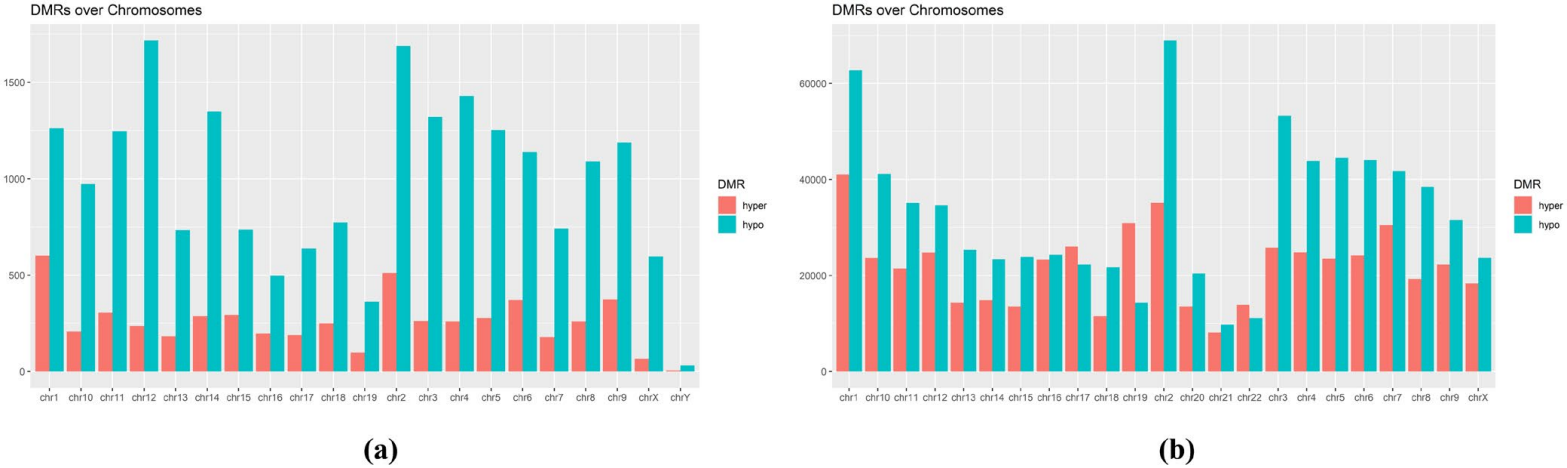
General analysis of differentially methylated patterns. (a) Distribution of DMRs on chromosomes in mouse group; (b) Distribution of DMRs on chromosomes in human group. (*X*‐axis: chromosome number; *Y*‐axis: Number of DMRs; Red bar: Hypermethylated DMRs; Green bar: hypomethylated DMRs.)

High‐throughput sequencing MBD‐seq data from ovarian tissue samples from three PNA mice and two control mice were preprocessed. After obtaining .*bam* files of methylation level in these ovarian tissue samples, genome‐wide differentially methylated region (DMR) analysis was performed with a screening threshold of fold ratio ≥ 2 and *p*‐value < 0.05. A total of 26 174 DMRs were obtained, of which 5411 were hypermethylated and 20 763 were hypomethylated (Tables S3, S4). There were significantly more hypomethylated regions than hypermethylated regions, and the proportion of hypomethylated regions on each chromosome was higher than that of hypermethylated regions (Figure 1a).

Similarly, the high‐throughput sequencing MBD‐seq data from three ovarian GC samples from PCOS patients and two control ovarian GC samples were preprocessed. Finally, 1 264 800 DMRs were screened, of which 504 605 were hypermethylated and 760 195 were hypomethylated. Figure 1b shows the distribution of hypermethylated and hypomethylated regions on each chromosome, showing that the proportion of hypermethylated regions on chromosomes 17 and 19 is higher than that of hypomethylated regions, but the proportion of hypomethylated regions on other chromosomes is higher than that of hypermethylated regions.

**FIGURE 2:**
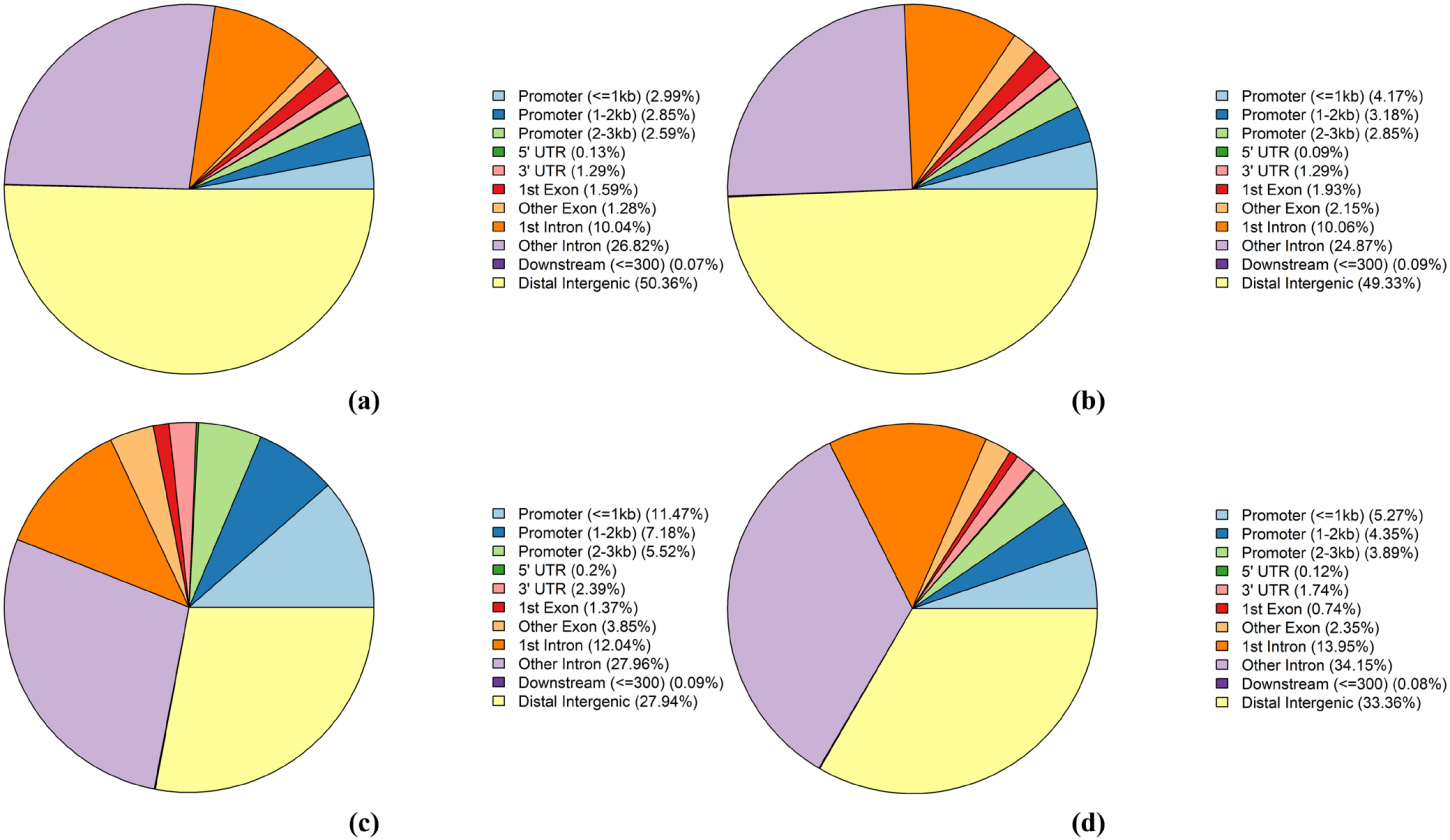
Analysis of differentially methylated patterns in different gene regions. (a) Hypermethylated region distribution in mouse group; (b) Hypomethylated region distribution in mouse group; (c) Hypermethylated region distribution in human group; (d) Hypomethylated region distribution in human group. (Color: Different gene regions.)

To further understand the distribution of DMRs in genomic characteristic regions, 26 174 DMRs were annotated and visualized statistically. As shown in Figure 2a,b, DMRs were mainly distributed in the distal intergenic region and intron region, including the first intron and other introns. A total of 2574 (9.83%) DMRs were located in the promoter region, including 456 (1.74%) hypermethylation regions and 2118 (8.09%) hypomethylation regions. There are much more hypomethylated regions than hypermethylated regions in the promoter region. For human, similarly, as shown in Figure 2c,d, DMRs were mainly distributed in the distal intergenic region and intron region. Compared with the mouse group, the proportion of DMRs located in the promoter region was significantly higher in the human group, and the number of hypermethylated regions was higher than hypomethylation regions in the promoter region.

**FIGURE 3:**
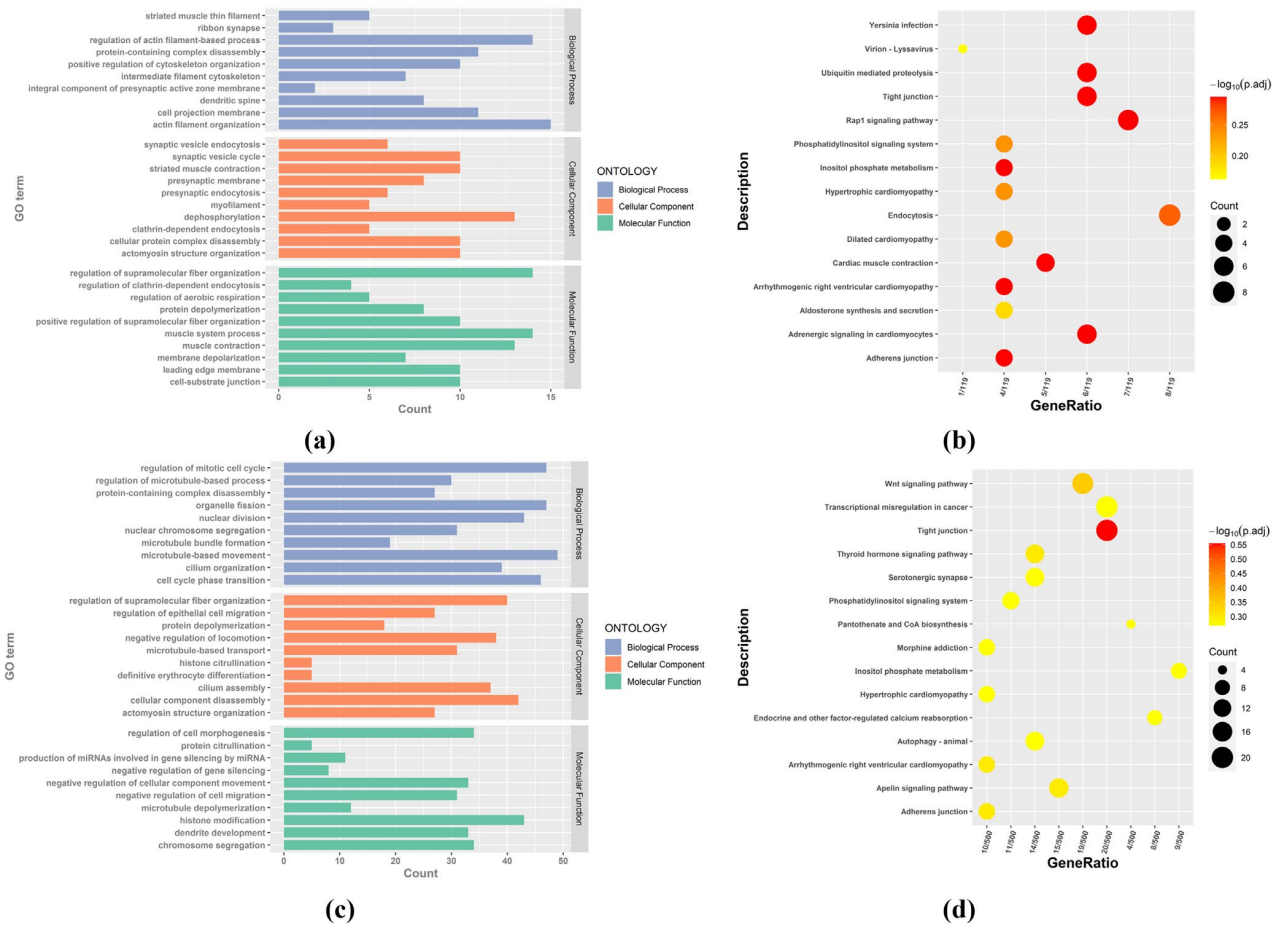
Enrichment analysis of DMGs' feature in mouse group. (a) and (b) GO analysis and KEGG analysis of promoter‐hypermethylation genes, respectively; (c) and (d) GO analysis and KEGG analysis of promoter‐hypomethylation genes, respectively. (For GO analysis, *X*‐axis: Gene counts in each GO term; *Y*‐axis: GO terms; Blue bar: Biological process; Orange bar: Cellular component; Green bar: Molecular function. For KEGG analysis, *X*‐axis: Gene ratio for each description; *Y*‐axis: Description in KEGG; Circular diameter: Gene counts; Color temperature: *p*‐value.)

We annotated DMRs and mapped 456 promoter regions with hypermethylation to 334 genes and 2118 promoter regions with hypomethylation to 1386 genes. In order to explore the function and significance of differentially promoter‐methylated genes (DMGs) in the process of PCOS, the promoter‐hypermethylation genes and the promoter‐hypomethylation genes were analyzed respectively (Figure 3). Gene Ontology (GO) and Kyoto Encyclopedia of Genes and Genomes (KEGG) analysis showed that the function of promoter‐differentially‐methylated genes is enriched in cell proliferation and migration, such as regulation of mitotic cell cycle (GO:0007346) and nuclear division (GO:0000280). It is mainly the promoter‐hypomethylation genes that are enriched in these biological pathways. The functions of these genes overlap with those of DEGs previously analyzed, suggesting that there is an interaction and co‐regulation of methylation levels and gene expression levels in the same pathway.

**FIGURE 4:**
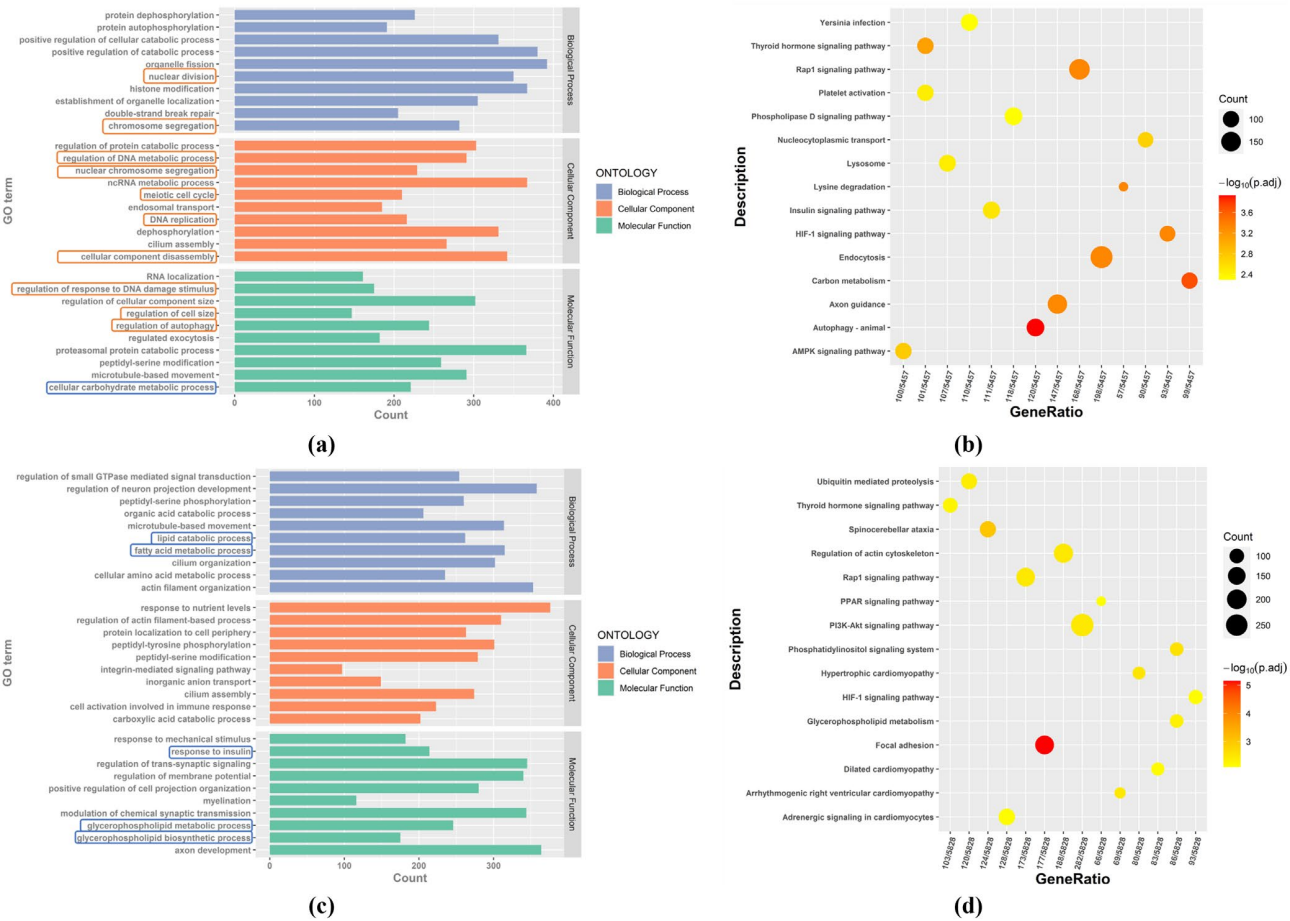
Enrichment analysis of DMGs' feature in human group. (a) and (b) GO analysis and KEGG analysis of promoter‐hypermethylation genes, respectively; (c) and (d) GO analysis and KEGG analysis; of promoter‐hypomethylation genes, respectively. (For GO analysis, *X*‐axis: Gene counts in each GO term; *Y*‐axis: GO terms; Blue bar: Biological process; Orange bars: Pathways related to cellular component; Green bar: Pathways related to molecular function; Orange box: Cell growth and proliferation; Blue box: Pathways related to metabolism. For KEGG analysis, *X*‐axis: Gene ratio for each description; *Y*‐axis: Description in KEGG; Circular diameter: Gene counts; Color temperature: −log_10_(*p*‐adjust).)

For human group, by annotating the DMRs, 121 946 promoter regions with hypermethylation were mapped to 17 843 genes, and 102 738 promoter regions with hypomethylation were mapped to 19 950 genes. The results of promoter‐hypermethylation gene function analysis are shown in (Figure 4a,b). The functions of promoter‐hypermethylation genes are mainly enriched in cell growth and proliferation, such as chromosome segregation (GO:0007059) and DNA replication (GO:0006260). In addition, they were also enriched in some pathways highly related to PCOS, such as cellular carbohydrate metabolic process (GO:0044262), insulin signaling pathway and thyroid hormone signaling pathway. The promoter‐hypomethylation genes were also functionally analyzed shown in (Figure 4c,d). They are mainly enriched in pathways closely related to PCOS, especially metabolic pathways, such as lipid catabolic process (GO:0016042) and triglyceride metabolic process (GO:0006650), as well as response to insulin (GO:0032868), and thyroid hormone signaling pathway.

#### 3.1.3 Analysis of Differentially Expressed miRNAs and Their Target Genes

**FIGURE 5:**
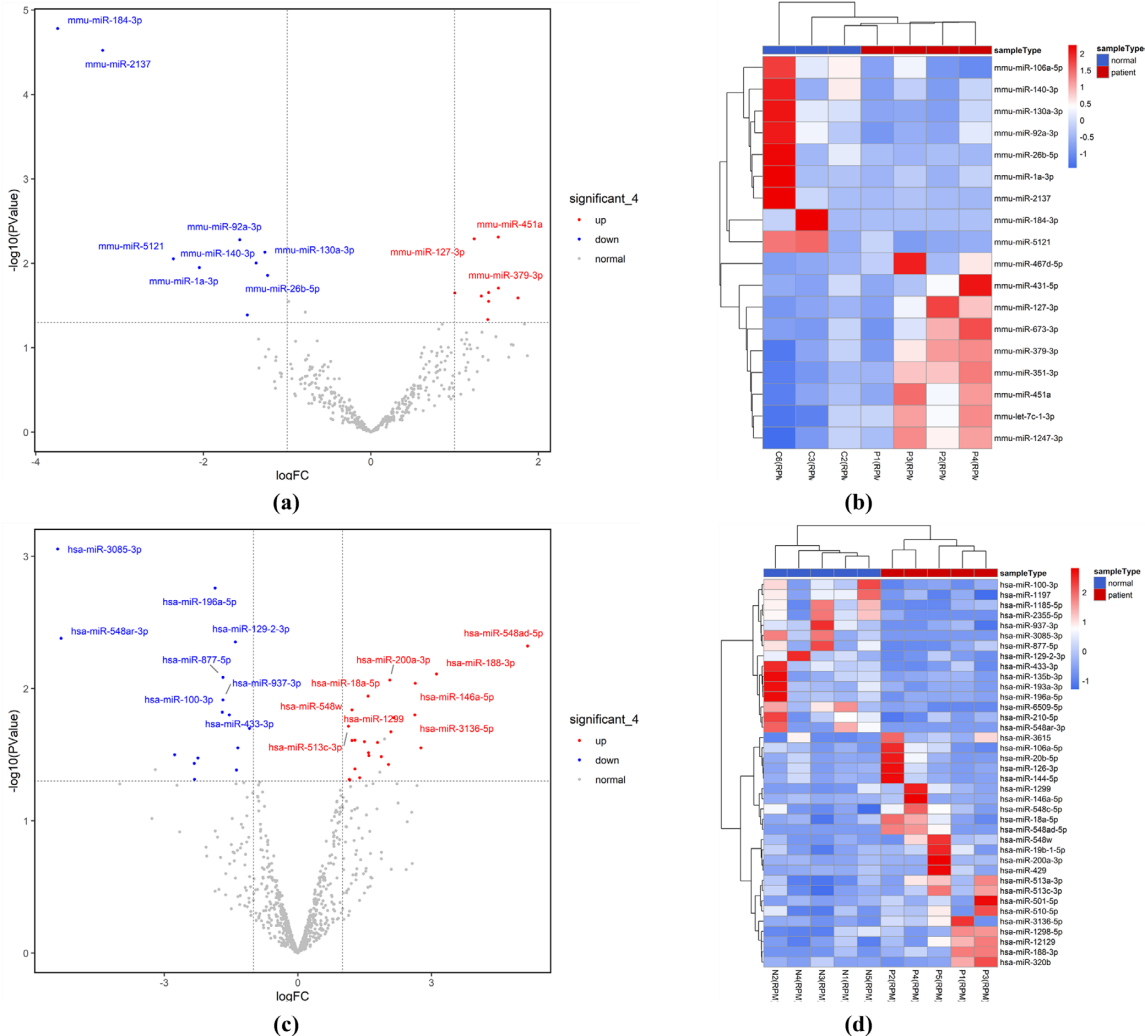
Results of differential analysis of DEmiRs. (a) Volcano plot of DemiRs in the mouse group; (b) Hierarchical clustering heat plot of DemiRs in the mouse group; (c) Volcano plot of DemiRs in the human group; (d) Hierarchical clustering heat plot of DemiRs in the human group. (For volcano maps, *X*‐axis: LogFC; *Y*‐axis: −log_10_(*p*‐value); Red dot: Upregulated miRNAs; Blue dot: Downregulated miRNAs; Gray dot: MiRNAs with no significant expression differences. For heatmaps, *X*‐axis: Sample type; *Y*‐axis: DEmiRs; Color temperature: Normalized DEmiR expression level.)

The miRNA‐seq data of ovarian tissue samples from four PNA mice and three control mice were preprocessed. After obtaining the miRNA expression matrix from the samples, differential analysis was performed and 18 differentially expressed miRNAs (DemiRs) were obtained, of which nine were upregulated and nine were downregulated (Table S5). Subsequently, hierarchical cluster analysis of DEmiRs was performed and the results were visualized using volcano plots and hierarchical clustering heat maps (Figure 5a,b) The classification between the PCOS and control groups was good, and there were significant differences in miRNA expression patterns in ovarian tissues.

For human group, the miRNA‐seq data of ovarian GC samples from five PCOS patients and five control ovarian GC samples were preprocessed. Thirty‐eight DEmiRs were obtained, of which 23 were upregulated and 15 were downregulated (Table S6). They will be used for subsequent multiomics joint analysis. Subsequently, a hierarchical cluster analysis of DEmiRs was performed, and the results were shown in Figure 5c,d, which indicated that the classification between the PCOS and control groups was as good as the mouse group.

#### 3.1.4 Analysis of Differentially Expressed Genes

**FIGURE 6:**
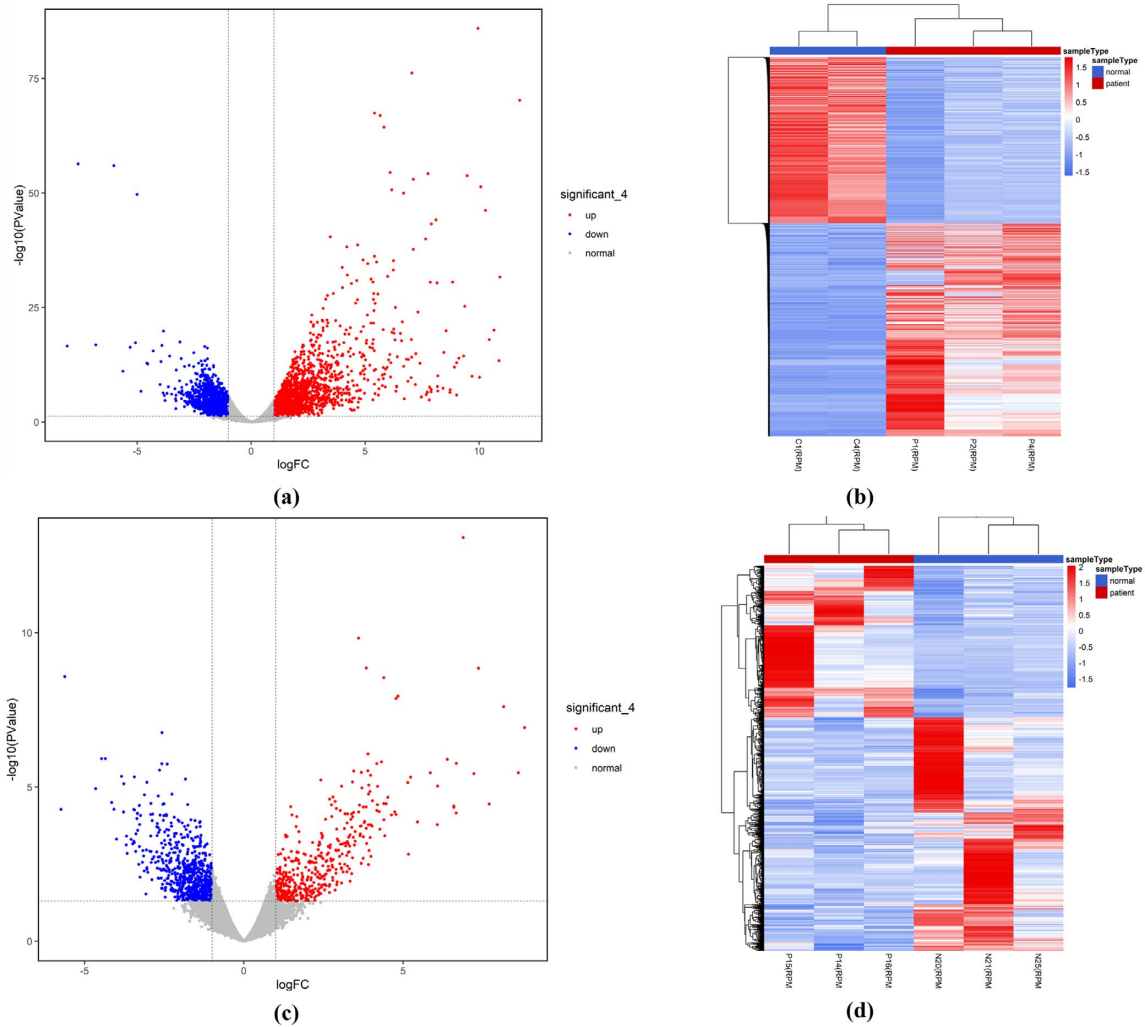
Results of differential analysis of DEGs. (a) Volcano plot of DEGs in the mouse group; (b) Hierarchical clustering heat plot of DEGs in the mouse group; (c) Volcano plot of DEGs in the human group; (d) Hierarchical clustering heat plot of DEGs in the human group. (For volcano maps, *X*‐axis: LogFC; *Y*‐axis: −log_10_(*p*‐value); Red dot: Upregulated DNAs; Blue dot: Downregulated DNAs; Gray dot: DNAs with no significant expression differences. For heatmaps, *X*‐axis: Sample type; *Y*‐axis: DEGs; Color temperature: Normalized DEG expression level.)

The high‐throughput sequencing RNA‐seq data of ovarian tissue samples from three PNA mice and two control mice were preprocessed, and the quality test results showed that the quality score for each base was above 30. Differential analysis revealed 3338 differentially expressed genes (DEGs), of which 1877 were upregulated and 1461 downregulated (Table S7). Hierarchical cluster analysis of DEGs was performed, and the results were visualized in (Figure 6a,b), which showed that the classification between the PNA group and the control group was good, and there were significant differences in the expression patterns of genes in ovarian tissues.

For human group, the high‐throughput sequencing RNA‐seq data of ovarian GC samples from three PCOS patients and three normal women were preprocessed. 1107 DEGs were obtained, of which 436 were upregulated and 671 were downregulated (Table S8). The results of hierarchical cluster analysis of DEGs were visualized using a volcano plot and a hierarchical cluster heat map (Figure 6c,d), showing that there were significant differences in the expression patterns of genes in ovarian tissues.

**FIGURE 7:**
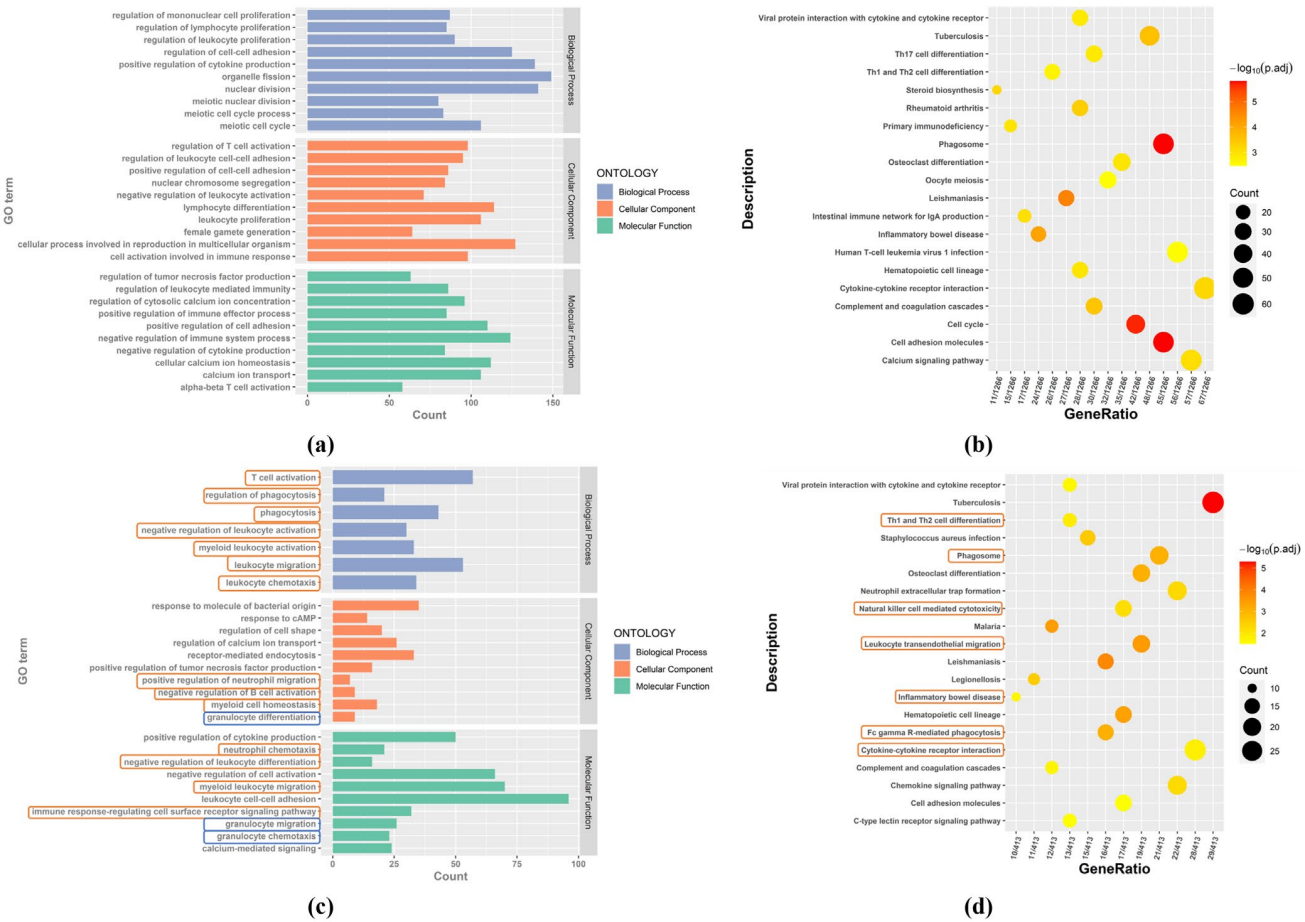
Results of enrichment analysis of DEGs. (a) Results of GO enrichment analysis of DEGs in the mouse group; (b) Results of KEGG enrichment analysis of DEGs in the mouse group; (c) Results of GO enrichment analysis of DEGs in the human group. (d) Results of KEGG enrichment analysis of DEGs in the human group. (For GO analysis, *X*‐axis: Gene counts in each GO term; *Y*‐axis: GO terms; Blue bar: Biological process; Orange bar: Cellular component; Green bar: Molecular function; Orange box: Cell growth and proliferation; Blue box: Pathways related to metabolism; Orange box: Pathways related to immune modulation; Blue box: Pathways related to GCs. For KEGG analysis, *X*‐axis: Gene ratio for each description; *Y*‐axis: Description in KEGG; Circular diameter: Gene counts; Color temperature: −log_10_(*p*‐adjust); Orange box: Pathways related to inflammation and immune response.)

In order to further study the regulatory effect of DEGs on PCOS, we conducted GO enrichment analysis and KEGG pathway analysis of DEGs to explore the biological processes and pathways related to PCOS. For the mouse group, we selected the top 10 *p*‐value entries in each category from the GO enrichment analysis shown in Figure 7a. It can be seen that the function of DEGs is mainly enriched in two aspects: cell proliferation (e.g., nuclear division, meiosis, cell cycle) and immune regulation (such as T cell activation, lymphocyte differentiation, cell activation in immune response). There are also GO entries related to the proliferation of immune cells, such as regulation of monocyte proliferation (GO:0032944) and regulation of lymphocyte proliferation (GO:0050670). Figure 7b shows the top 20 *p*‐value results for the KEGG pathway, indicating that DEGs are involved in pathways highly associated with PCOS, such as steroid biosynthesis and oocyte meiosis, as well as pathways related to inflammation and immune response, such as Th1 and Th2 cell differentiation, phagosome, cytokine‐cytokine receptor interaction. It is also involved in cell proliferation pathways such as cell cycle.

Figure 7c shows the results of GO enrichment analysis for the human group. The function of DEGs is mainly enriched in immune regulation and GC‐related biological processes. There are many pathways related to immune modulation, such as T cell activation (GO:0042110) and leukocyte migration (GO:0050900). The biological processes related to GCs are also closely related to the occurrence and development of PCOS, mainly focusing on GC differentiation (GO:0030851), granulocyte migration (GO:0097530), and chemotaxis of GCs (GO:0071621). Figure 7d shows the results of KEGG pathway analysis, showing that DEGs are also involved in pathways related to inflammation and immune response, such as Th1 and Th2 cell differentiation, phagosome, and cytokine‐cytokine receptor interaction.

### 3.2 Combined Analysis of Epigenomics and Transcriptomics

#### 3.2.1 Combined Analysis of the Methylome With the Transcriptome

**FIGURE 8:**
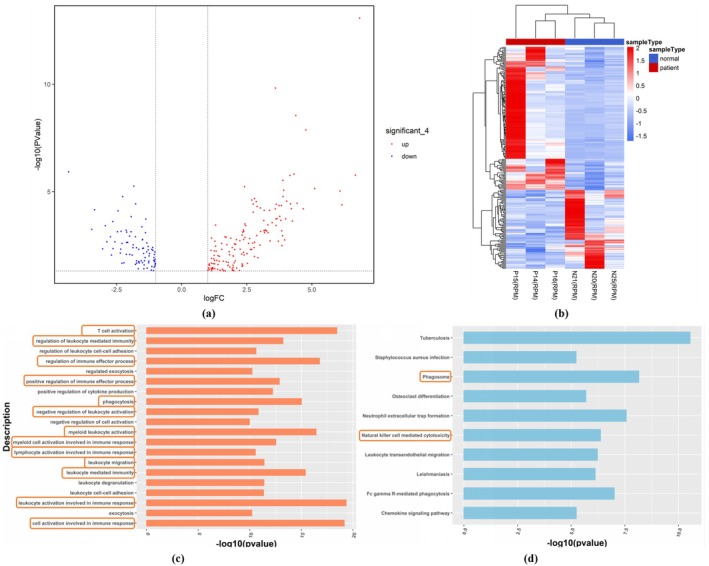
Mouse and human homologous DEG analysis. (a) Volcano plot (*X*‐axis: LogFC; *Y*‐axis: −log_10_(*p*‐value); Red dot: Upregulated DNAs; Blue dot: Downregulated DNAs); (b) Hierarchical clustering heat map (*X*‐axis: Sample type; *Y*‐axis: Homologous DEGs; Color temperature: Normalized homologous DEG expression level); (c) GO functional analysis (*X*‐axis: −log_10_(*p*‐value) in each GO description; *Y*‐axis: GO descriptions; Orange box: Pathways related to immunity and inflammation); (d) KEGG functional analysis (*X*‐axis: −log_10_(*p*‐value) in each KEGG description; *Y*‐axis: KEGG descriptions; Orange box: Pathways related to immunity and inflammation.)

To further investigate the regulatory mechanism of methylation in PCOS, we combined the results of RNA‐seq and MBD‐seq data analysis. First, we combined 3338 DEGs screened from the PNA mouse model group and 1107 DEGs screened from the clinical PCOS patient group. The R‐package homologene was used to find out the human homologous genes corresponding to the DEGs in the mouse group and then intersected with the DEGs in the human group. We finally obtained 236 DEGs, of which 152 were upregulated and 84 were downregulated. Hierarchical clustering analysis was performed, and the final results were visualized in Figure 8a,b. It can be seen that the PCOS group and the control group were well classified, and there were significant differences in the expression profile of DEGs.

We performed GO enrichment analysis and KEGG pathway analysis to explore the role of DEGs in the occurrence and development of PCOS. Figure 8c,d showed the top 20 and top 10 entries with *p*‐values in the GO analysis and KEGG analysis, respectively. The function of DEGs is mainly focused on immunity and inflammation, such as T cell activation (GO:0042110), leukocyte‐mediated immune regulation (GO:0002703), and immune effector regulation (GO:0002697).

Subsequently, we combined 1720 DMGs from the PNA mouse model group with 120 581 DMGs from the PCOS patient group using similar methods. Finally, 1211 DMGs were obtained. We screened the corresponding DEGs and DMGs in the RNA‐seq group and MBD‐seq group, respectively, and then carried out transcriptome‐methylome multiomics analysis in order to further explore their interactions in PCOS regulation. After obtaining 236 DEGs and 1211 DMGs, the two groups of genes were intersected and screened according to the criteria of negative regulation between methylation level and expression level in the human group. 10 promoter‐hyper‐methylation & hypo‐expression genes and 16 promoter‐hypo‐methylation & hyper‐expression genes were obtained (Table 3). Finally, we screened genes with the same trend for differential expression in mouse and human groups. One gene, *ANXA1*, with promoter‐hypomethylation and high expression was obtained. Most genes exhibit genetic or epigenetic differences between mice and humans. For instance, *CD300A*, *KCNMA1*, and *SORBS1* exhibited upregulated expression in both mouse and human PCOS phenotypes. However, their promoter methylation trends were opposite between the two species: hypermethylation was detected in PNA mice, while hypomethylation was detected in PCOS patients.

**TABLE 3:** Genes whose methylation levels are inversely correlated with gene expression levels.

Gene	Log_2_FC	*p*	Methylation	Expression
*C1orf159*	−1.028	0.00654	Hyper	Down
*COL7A1*	−1.067	0.01586	Hyper	Down
*MAP7D2*	−2.929	0.00039	Hyper	Down
*OBSCN*	−1.337	0.00247	Hyper	Down
*OTOF*	−1.286	0.00517	Hyper	Down
*RAP1GAP*	−1.145	0.03085	Hyper	Down
*RBM47*	−1.016	0.02427	Hyper	Down
*SCARB1*	−1.734	0.01669	Hyper	Down
*SLIT3*	−2.126	0.00842	Hyper	Down
*VWF*	−1.993	0.00852	Hyper	Down
*ANXA1*	2.029	0.00271	Hypo	Up
*CCDC68*	2.005	0.04848	Hypo	Up
*CD14*	1.940	0.00401	Hypo	Up
*CD300A*	3.392	0.00198	Hypo	Up
*CYTH4*	3.377	0.00007	Hypo	Up
*EPHA2*	1.537	0.00258	Hypo	Up
*FCGR3A*	3.738	0.00004	Hypo	Up
*FCGR3B*	3.916	0.00001	Hypo	Up
*GNPDA1*	1.053	0.02547	Hypo	Up
*IL6R*	1.210	0.02266	Hypo	Up
*ITGAX*	2.411	0.00001	Hypo	Up
*KCNMA1*	1.150	0.04834	Hypo	Up
*LGALS3*	1.249	0.00578	Hypo	Up
*PADI2*	4.433	0.00003	Hypo	Up
*PTK2B*	1.302	0.00346	Hypo	Up
*SORBS1*	1.060	0.02346	Hypo	Up

#### 3.2.2 Combined Analysis of the miRNAs With the Transcriptome

miRNAs negatively regulate the expression of target genes by inhibiting or degrading the translation of target mRNAs. To further investigate the regulatory mechanisms of miRNAs in PCOS, we performed a combined analysis of miRNA‐seq and RNA‐seq data. First, we aligned 18 DEmiRs screened from the PNA mouse model group to human homologous genes, then intersected with 38 DEmiRs screened from the PCOS patient group, and finally obtained 1 DEmiR: hsa‐miR‐106a‐5p. We predicted the target genes of has‐miR‐106a‐5p, intersected them with the final 236 DEGs, screened them according to the criteria of negative regulation between miRNA expression levels and gene expression levels, and finally obtained four differentially expressed target genes in PCOS patients: *LDLR*, *RUNX1*, *FICD*, and *STAC2* (Table 4). We screened genes with the same trend for differential expression in mouse and human groups and *LDLR* with low expression was obtained.

**TABLE 4:** Differential expression of hsa‐miR‐106a‐5p and its target genes.

DEmiR	Expression	DEG	Expression	Log_2_FC	*p*
hsa‐miR‐106a‐5p	Up	*LDLR*	Down	−1.164	0.04553
		*RUNX1*	Down	−1.149	0.00486
		*FICD*	Down	−1.058	0.03297
		*STAC2*	Down	−2.109	0.00077

Abbreviations: DEG, differentially expressed gene; DEmiR, differentially expressed microRNA.

### 3.3 Validation of Differentially Expressed Genes

**FIGURE 9:**
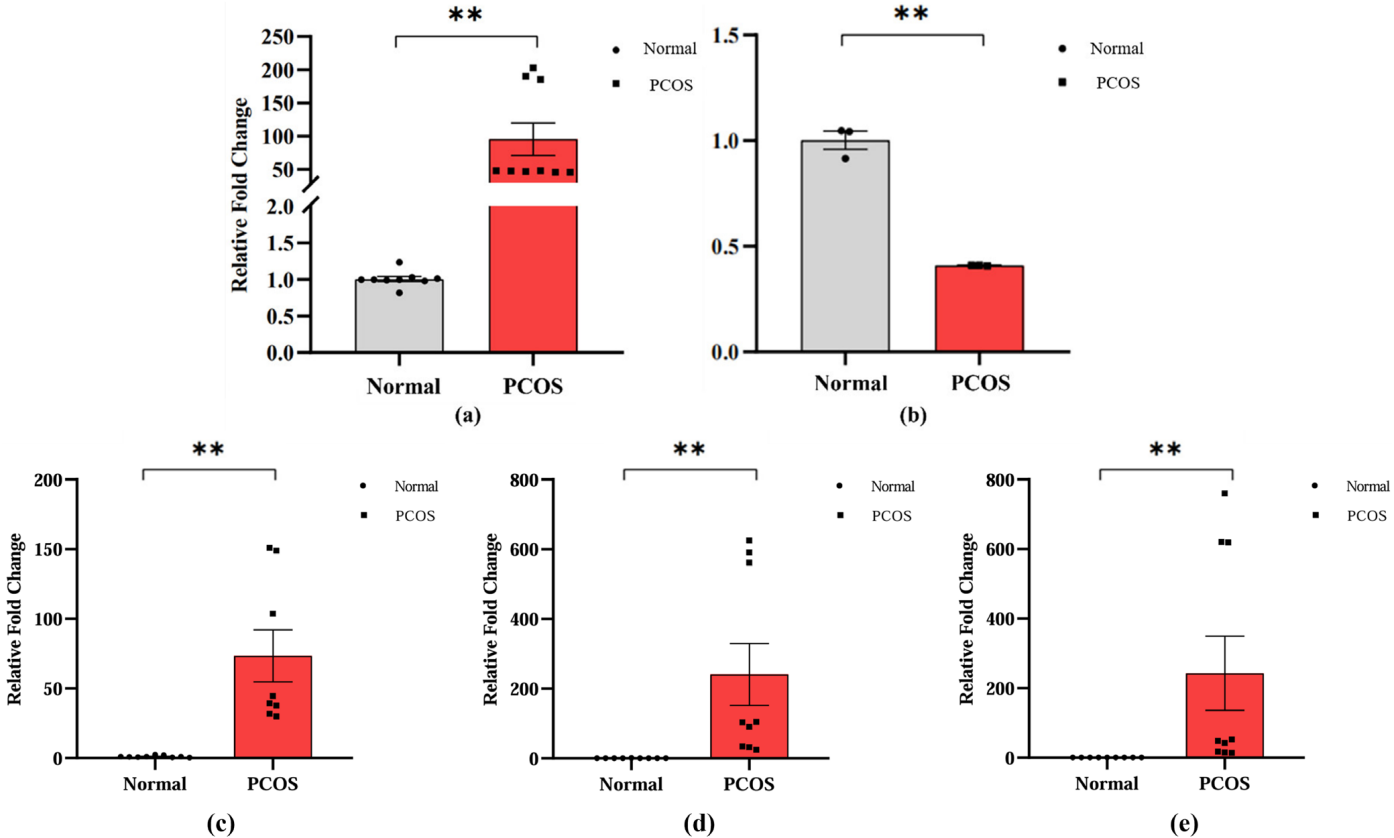
PCR validation results for human. (a) PCR results for *ANXA1*; (b) PCR results for *LDLR*; (c) PCR results for *CD300A*; (d) PCR results for *KCAMN1*; (e) PCR results for *SORBS1*. (*X*‐axis: Sample type; *Y*‐axis: Relative fold change; Circle dot: Normal samples; Square dot: PCOS samples; **p‐value < 0.01.)

The results of PCR validation are shown in Figure 9. *ANXA1* was significantly differentially expressed in PCOS patients (Figure 9a), which was consistent with the above data analysis results with high reliability. For *LDLR*, there was also a significant differential expression (Figure 9b), matching the results of the previous analysis. Therefore, both genes were included in the later discussion. We also conducted PCR validation in human PCOS patient samples for *CD300A*, *KCNMA1*, and *SORBS1* shown in Figure 9c–e. In PCOS patients, their expression is significantly upregulated.

### 3.4 Metabolomic Analysis of Follicular Fluid in Patients With PCOS

Combined with the *p*‐value of the independent‐sample t‐test, the FC value of the fold analysis method and the VIP value of the OPLS‐DA model (Figures [Link], [Link]), *p*‐value < 0.05, FC > 2 or < 0.5, and VIP value > 1 were used to identify candidate differential lipids. 34 significantly changed follicular fluid lipid metabolites (as shown in Table 5) were jointly screened and used as potential biomarkers for PCOS for subsequent discussion. In follicular fluid positive ion samples, 16 lipids, including triglyceride (TG) (16:0/17:0/18:1), TG (18:0/18:0/18:1), and TG (16:0/14:0/16:1), were significantly increased in the follicular fluid of PCOS patients. 10 lipids, including TG (8:0/8:0/8:0), TG (8:0/10:0/10:0), and TG (10:0/12:0/14:0), were significantly decreased. In follicular fluid negative ion samples, only phosphatidylinositol (PI) (16:0/16:1) was significantly upregulated in the PCOS group, while the contents of seven lipids, including phosphatidylcholine (PC) (16:0/22:5) and phosphatidylethanolamine (PE) (16:1e/22:6), were significantly downregulated.

**TABLE 5:** Follicular fluid metabolomics differential analysis results.

Ion model	Metabolite	VIP score	*p*	Log_2_FC	Expression
ESI+	TG(16:0/17:0/18:1)	1.614747	0.023584	2.124	Up
ESI+	TG(18:0/18:0/18:1)	1.699763	0.001321	2.3058	Up
ESI+	TG(16:0/14:0/16:1)	1.562229	0.035085	2.4569	Up
ESI+	TG(8:0/8:0/8:0)	1.659041	0.003977	0.4439	Down
ESI+	TG(8:0/10:0/10:0)	1.657744	0.007634	0.018469	Down
ESI+	TG(10:0/12:0/14:0)	1.623083	0.009687	0.09831	Down
ESI+	TG(12:0/12:0/14:0)	1.638778	0.006995	0.047251	Down
ESI+	TG(16:0/12:0/18:2)	1.525541	0.040716	2.4476	Up
ESI+	TG(12:0/18:2/18:2)	1.624639	0.0166	2.2911	Up
ESI+	PI(18:0/18:2)	1.57181	0.014783	0.41856	Down
ESI+	TG(18:0/18:0/20:4)	1.699763	0.001321	2.3058	Up
ESI+	TG(8:0/12:0/14:0)	1.591713	0.012031	0.11449	Down
ESI+	TG(16:0/16:0/16:0)	1.520427	0.021398	2.1631	Up
ESI+	TG(16:0/14:0/18:1)	1.658743	0.013569	2.4973	Up
ESI+	TG(16:0/14:0/18:2)	1.629764	0.019567	2.4078	Up
ESI+	TG(12:0/18:2/20:5)	1.624639	0.0166	2.2911	Up
ESI+	TG(18:0/16:0/20:4)	1.782824	0.001793	2.5309	Up
ESI+	TG(18:0/16:0/16:0)	1.523474	0.017759	2.0329	Up
ESI+	TG(16:0/12:0/12:0)	1.666418	0.006766	0.073958	Down
ESI+	TG(16:0/16:0/18:1)	1.61747	0.008523	2.2562	Up
ESI+	TG(16:0/16:1/18:1)	1.583	0.011655	2.1568	Up
ESI+	TG(18:0/16:0/18:1)	1.782824	0.001793	2.5309	Up
ESI+	TG(8:0/8:0/10:0)	1.64501	0.009041	0.02533	Down
ESI+	TG(8:0/12:0/12:0)	1.623154	0.009988	0.054346	Down
ESI+	PE(16:0p/22:6)	1.389991	0.029414	0.37848	Down
ESI+	TG(18:1/12:0/18:2)	1.609866	0.028858	2.1423	Up
ESI—	PI(16:0/16:1)	1.754602	0.040597	2.0594	Up
ESI—	PC(18:1e/22:6)	1.767388	0.031395	0.49872	Down
ESI—	PC(18:1/22:5)	1.66684	0.047324	0.48628	Down
ESI—	PC(16:0/22:5)	2.0063	0.011515	0.43297	Down
ESI—	PE(16:1e/22:6)	1.869659	0.019478	0.24385	Down
ESI—	PI(18:0/18:2)	1.994194	0.013502	0.40203	Down
ESI—	PC(18:1/20:3)	2.138099	0.005881	0.26951	Down
ESI—	PE(18:2e/22:6)	1.747239	0.030516	0.34552	Down

Abbreviations: ESI, electrospray ionization; PC, phosphatidylcholine; PE, phosphatidylethanolamine; PI, phosphatidylinositol; TG, triglyceride.

## 4 Discussion

### 4.1 An Overview of Combined Analysis

In this study, the enrichment analysis of GO and KEGG was plotted in the data analysis of each omics. It can be seen that DMGs are mainly enriched in cell growth and proliferation and lipid metabolism pathways, while DEGs are mainly enriched in immune regulation‐related pathways.

After that, we took the homologous genes of mice and humans. Then we looked for DMGs and DEGs common in both species and took the intersection of them. The number of genes obtained after screening was drastically reduced and mainly enriched in metabolism‐ and inflammation‐related pathways. Similar results were obtained for the analysis of miRNA expression, with a sharp decrease in the number after taking the intersection. It is evident that some genes play important roles in PCOS across species as well as across omics and are ideal for study.

### 4.2 The Regulatory Role of DNA Methylation in PCOS

DNA methylation is closely related to PCOS. The changes in genomic methylation status can affect gene expression, which in turn plays a role in the occurrence and development of polycystic ovary syndrome. Approximately 50 genes with abnormal methylation status are associated with PCOS [26]. These genes are associated with symptoms of PCOS, such as insulin resistance [27], ovulation regulation, [28] hyperandrogenism [29], and a range of concurrent cancers [30].

*ANXA1* plays an important role in the negative regulation of innate immunity. It can inhibit the migration and recruitment of neutrophils at the site of inflammation, thereby reducing their pro‐inflammatory activity. Otherwise, severe inflammatory lesions may occur in the tissue [31]. Glucocorticoids are also involved in this process, promoting the anti‐inflammatory effects of *ANXA1* [32]. *ANXA1* can also promote neutrophil apoptosis and macrophage cellular behavior to clear necrotic neutrophils. These cells or cell fragments activate the body's adaptive immune system, and *ANXA1* can shorten their exposure time, limiting their stimulation of the immune system [33]. Meanwhile, *ANXA1* is able to inhibit pro‐inflammatory Toll‐like receptors in dendritic cells, giving them a degree of tolerance [34]. The effect of *ANXA1* on T cells is still inconclusive. Under normal conditions, the expression level of *ANXA1* gene in T cells is low, but it is upregulated in inflammatory environments [35]. Studies have shown that when T cells are stimulated, the expression of *ANXA1* receptors is also upregulated [36]. Inflammatory biomarkers play a crucial role in regulating ovarian function. Any disturbances related to inflammation may lead to ovarian dysfunction, thereby contributing to the development of PCOS [37]. Therefore, the upregulation of *ANXA1*, which possesses anti‐inflammatory properties, could be one of the body's adaptive responses to the onset of PCOS.

Aberrant hypermethylation of *ANXA1* is associated with its aberrant expression and a variety of cancers. Compared with the control group, the methylation level of the *ANXA1* gene in nasopharyngeal carcinoma tissues was extremely high, reaching 92%. This may be a direct cause of the decline in *ANXA1* expression in nasopharyngeal carcinoma, accelerating lymph node metastasis in nasopharyngeal carcinoma. The expression level of *ANXA1* in B‐cell lymphoma, prostate cancer, and esophageal cancer was also significantly decreased [38].

Current research focuses on the effects of hypermethylation and low expression of *ANXA1* on cancer occurrence and progression. In this study, we found that *ANXA1* showed hypomethylation in the promoter region and high expression in PCOS patients. *ANXA1* antagonizes PPARγ signaling in the peroxisome proliferator‐activated receptor (PPAR) pathway. PPARγ inhibition in adipocytes can reduce insulin sensitivity [39] which may be linked to the symptoms of IR in PCOS. However, it is worthy to note that none of the samples of PCOS patients in this study met the criteria for obesity (BMI > 30). Considering the role of the gene *ANXA1* in metabolism and inflammation, future studies need to include samples of obese PCOS patients. This may make the role of *ANXA1* more visible. On the other hand, the selection of non‐obese women enables this study to avoid the possible interference caused by obesity factors, which has certain reference value. Meanwhile, studies have shown that *ANXA1* is associated with intrauterine growth restriction (IUGR) in rat models. IUGR can increase the risk of developing type 2 diabetes, which aligns with the frequent occurrence of insulin resistance symptoms in PCOS patients [40].

However, many homologous genes do not exhibit entirely consistent patterns of gene expression and methylation changes, such as *CD300A*, *KCNMA1*, and *SORBS1*. These genes show upregulated expression in both mouse and human PCOS phenotypes. Yet, their promoter methylation trends are opposite between the two species: hypermethylation is observed in PNA mice, while hypomethylation is detected in PCOS patients. We believe this reflects species‐specific differences between mice and humans. Additionally, it suggests that the expression of these genes may be regulated by more complex mechanisms, where promoter methylation might serve as merely one contributing factor—or potentially play no role at all, which requires further research to elucidate.

### 4.3 The Regulatory Role of miRNA in PCOS

Altered miRNA expression has a significant impact on the development of various diseases. There has been sufficient evidence to support the combination between miRNA dysregulation in GCs and PCOS, which can be used to identify potential therapeutic targets [41]. Dysregulation of miRNAs in follicular fluid may be one of the key factors in ovulatory dysfunction in women with PCOS [42]. Also, miRNAs affect many biological processes including glucose and lipid metabolism; thus, they play an important role in insulin signaling pathway and IR in PCOS [43].

In this study, we found that one miRNA, hsa‐miR‐106a‐5p, had a higher expression in PCOS patients. Some studies have confirmed that hsa‐miR‐106a‐5p is significantly associated with renal cell carcinoma (RCC) progression and may serve as a candidate biomarker and a novel therapeutic target to inhibit RCC metastasis [44]. It has also been found that hsa‐miR‐106a‐5p is highly expressed in lung adenocarcinoma (LUAD), regulates the phosphorylation of the AMPK pathway, and is involved in the proliferation, migration, and autophagy of LUAD cells [45]. This may indicate that miR‐106a‐5p is associated with multiple cancer complications in PCOS.

This present study found significantly reduced *LDLR* expression in patients with PCOS, which is consistent with existing studies. The specific reduced expression of adipose LDLR in PCOS may be associated with hyperandrogenism, which may be related to the tendency of PCOS patients with concomitant liver disease to progress to non‐alcoholic steatohepatitis (NASH) [46]. Meanwhile, the protein convertase PCSK9 promotes LDLR degradation in hepatocytes, and its expression is elevated in patients with PCOS. The high expression of PCSK9 may have an inhibitory effect on both mRNA and a protein expression level of *LDLR* [47]. LDLR deficiency in cells leads to reduced estrogen biosynthesis and secretion, affecting follicular cell proliferation and its steroid hormone synthesis [48]. LDLR is a key enzyme that regulates lipid metabolism, and abnormalities in metabolic status may negatively impact the female reproductive system and contribute to obesity (manifested as elevated BMI). Therefore, the downregulation of *LDLR* expression may be associated with the development and progression of PCOS.

### 4.4 The Regulatory Role of Lipid Metabolites in PCOS

Studies have shown that the phosphoinositide‐3‐kinase–AKT (PI3K‐AKT) pathway is linked to common symptoms of PCOS, including insulin resistance (IR), obesity, and polycystic follicles [49]. The upregulation of PI may be associated with abnormalities in the PI3K‐AKT pathway in PCOS. During the human metabolic process, insulin binding to its receptor initiates the PI3K‐AKT pathway to regulate glucose metabolism. Specifically, activated PI3K phosphorylates phosphatidylinositol‐4,5‐bisphosphate (PIP2) to generate phosphatidylinositol‐3,4,5‐triphosphate (PIP3). PIP3 indirectly activates AKT, which mediates most key metabolic effects of insulin. As PIP2 is derived from phosphorylation of PI, elevated PI levels may suggest impaired PIP2 production, thus causing reduced PIP3 generation and then failure of AKT activation, finally leading to disrupted insulin‐mediated metabolic functions, aligning with the IR symptom commonly observed in PCOS [50].

The expression of PE in the follicular fluid of PCOS patients decreased. Studies have shown that PE levels are negatively correlated with the free androgen index (FAI). FAI is closely related to hyperandrogenemia, a classic symptom of PCOS. Additionally, FAI can serve as a potential marker for insulin resistance, further supporting the association between PE and PCOS [51].

### 4.5 Conclusions

In this study, we jointly analyzed the genome‐wide RNA‐seq, MBD‐seq and miRNA‐seq sequencing data of ovarian tissues from PNA mouse models and GCs from PCOS clinical patients. We explored the regulatory mechanism of DNA methylation and miRNA in PCOS, the potential miRNAs and genes as diagnostic markers for PCOS, and their biological functions. Two genes (*ANXA1* and *LDLR*) were screened out, which play an important role in the PCOS‐related pathways.

We screened out potential biomarkers for PCOS diagnosis and elucidated the regulatory pathways involved in them, which also had suggestive significance for the therapeutic targets of PCOS. Combined with the results of multiomics detection in clinical patients, the mechanism of PCOS occurrence and development can be further elaborated, so as to provide a more solid theoretical basis for the development of diagnostic kits and targeted treatment regimens for PCOS. However, the small number of samples used in this study may result in less reliable results. In future studies, we can further increase the number of samples and select PCOS patient samples that better reflect the heterogeneity of PCOS (including but not limited to obesity). In practical application, doctors can collect micro blood samples from subjects for high‐throughput sequencing. The sequencing results will be analyzed for differential expression. If *ANXA1* has a significantly high expression, and the expression of *LDLR* is found low, the subject can be considered to be at a higher risk of developing PCOS, achieving the purpose of early diagnosis of PCOS.

## Ethics Statement

This study has been approved by the Human Research Ethics Committee of Yuncheng Central Hospital (Approval number: KYLL2019073). The animal experiments performed in the study were approved by the Experimental Animal Welfare & Ethics Committee of the School of Biomedical Engineering, Shanghai Jiao Tong University (No. 2019005), and conform to the animal care and use guidelines of Shanghai Jiao Tong University Experimental Animal Center. All procedures followed were in accordance with t the Helsinki Declaration of 1964 and its later amendments. Animal Studies: The mice used in the study were purchased from the Institute of Cancer Research (ICR) Animal Experimental Center of Yangzhou University. The mice were housed in the Drum Tower Hospital Animal Experimental Center with constant temperature, humidity, and light, and had free access to water and food. All of the animal experiments in this study followed the guidelines of the Laboratory Animal Control Committee (Jiangsu Province, China).

## Consent

Participants (17 PCOS patients and 17 normal subjects) were recruited in Yuncheng Central Hospital of Shaanxi Province. All of them agreed to participate in the study and provided written informed consent.

## Conflicts of Interest

The authors declare no conflicts of interest.

## Supporting information


**Figure S1.** The result of MSP.

**Figure S2.** Analysis of follicular fluid positive ion PCA results.

**Figure S3.** Analysis of follicular fluid negative ion PCA results.

**Figure S4.** Results of multivariate statistical analysis of metabolomics of follicular fluid positive ions.

**Figure S5.** Results of multivariate statistical analysis of metabolomics of follicular fluid negative ions.

**Table S1.** Primers used for MSP.

**Table S2.** Primers used for PCR.

**Table S3.** Hypermethylated DMRs in mouse group.

**Table S4.** Hypomethylated DMRs in mouse group.

**Table S5.** DemiRs in mouse group.

**Table S6.** DemiRs in human group.

**Table S7.** DEGs in mouse group.

**Table S8.** DEGs in human group.

## Data Availability

RNA‐seq and MBD‐seq of ovarian tissues of PNA mice: GEO Accession viewer (nih.gov). miRNA‐seq in ovarian tissues of PNA mice: GEO Accession viewer (nih.gov). Identification of epigenetic interactions between miRNA and DNA methylation associated with polycystic ovarian syndrome: GEO Accession viewer (nih.gov).
