# Supplementary figures and images for: Combined Multiomics Analysis Reveals the Role of ANXA1 Methylation and miRNA‐Targeted LDLR in Polycystic Ovary Syndrome
Source: Reprod Med Biol. 2025 Jun 23;24(1):e12664. doi: 10.1002/rmb2.12664 (PMC12183603; doi:10.1002/rmb2.12664)

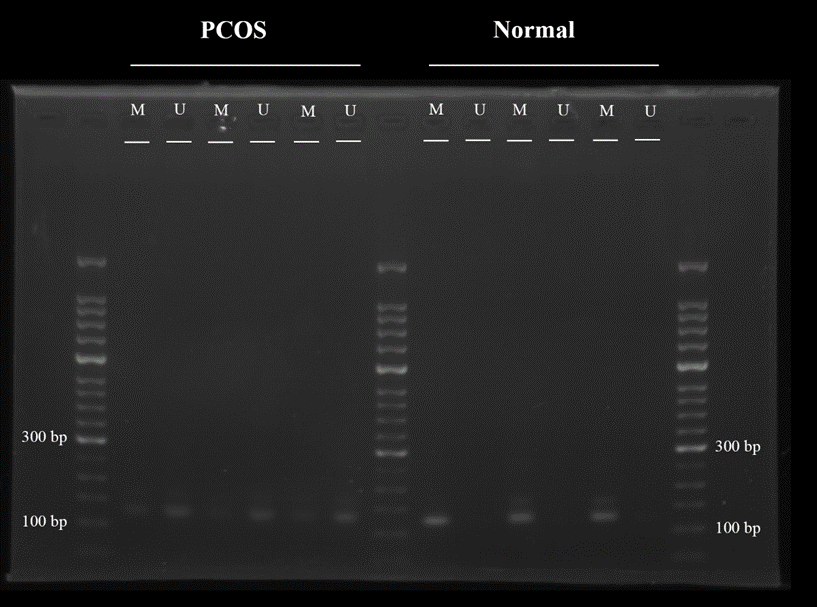

Supplement: Supplementary file 1 — Figure S1. The result of MSP. [file RMB2-24-e12664-s011.tif]

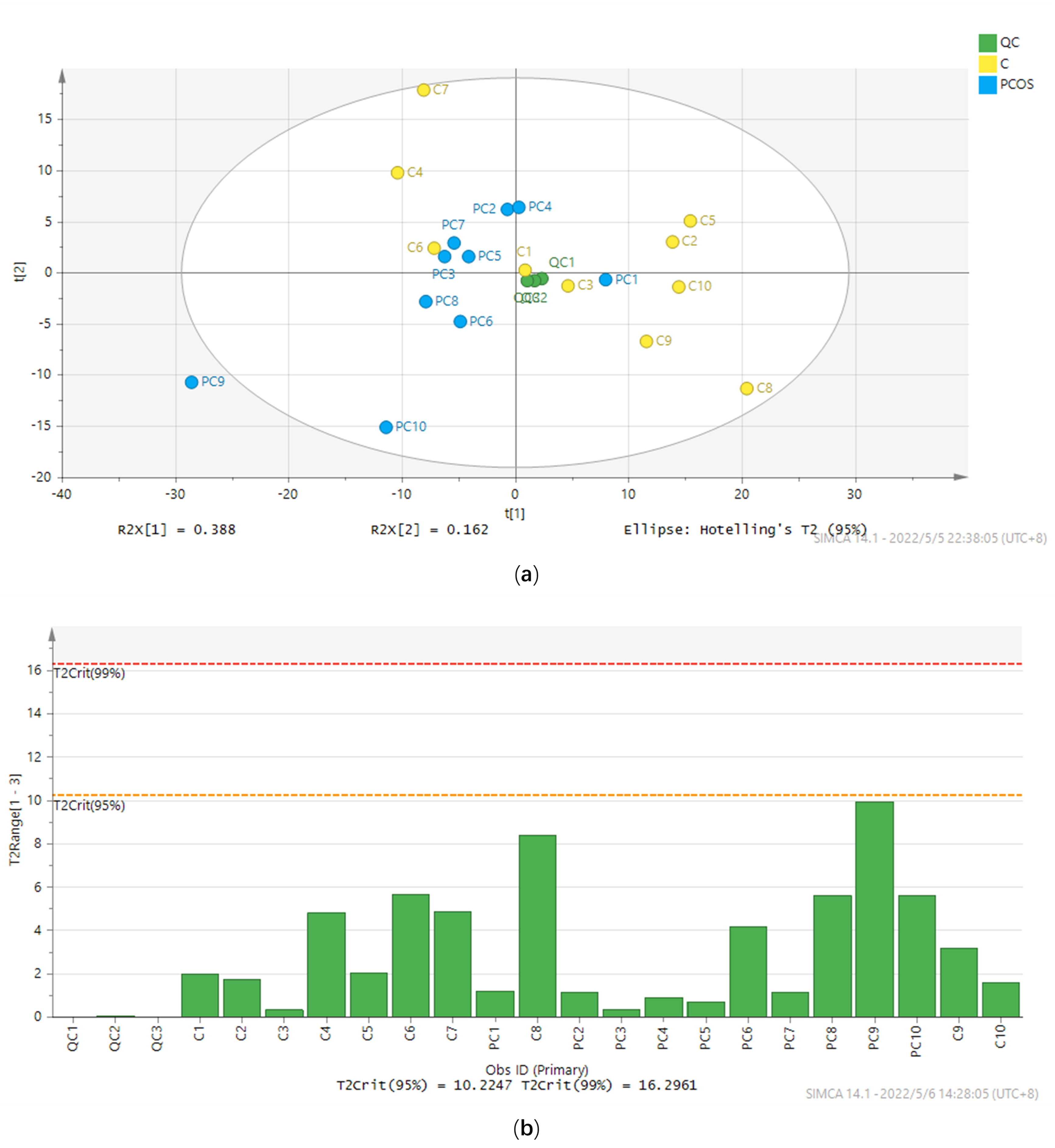

Supplement: Supplementary file 2 — Figure S2. Analysis of follicular fluid positive ion PCA results. [file RMB2-24-e12664-s001.tif]

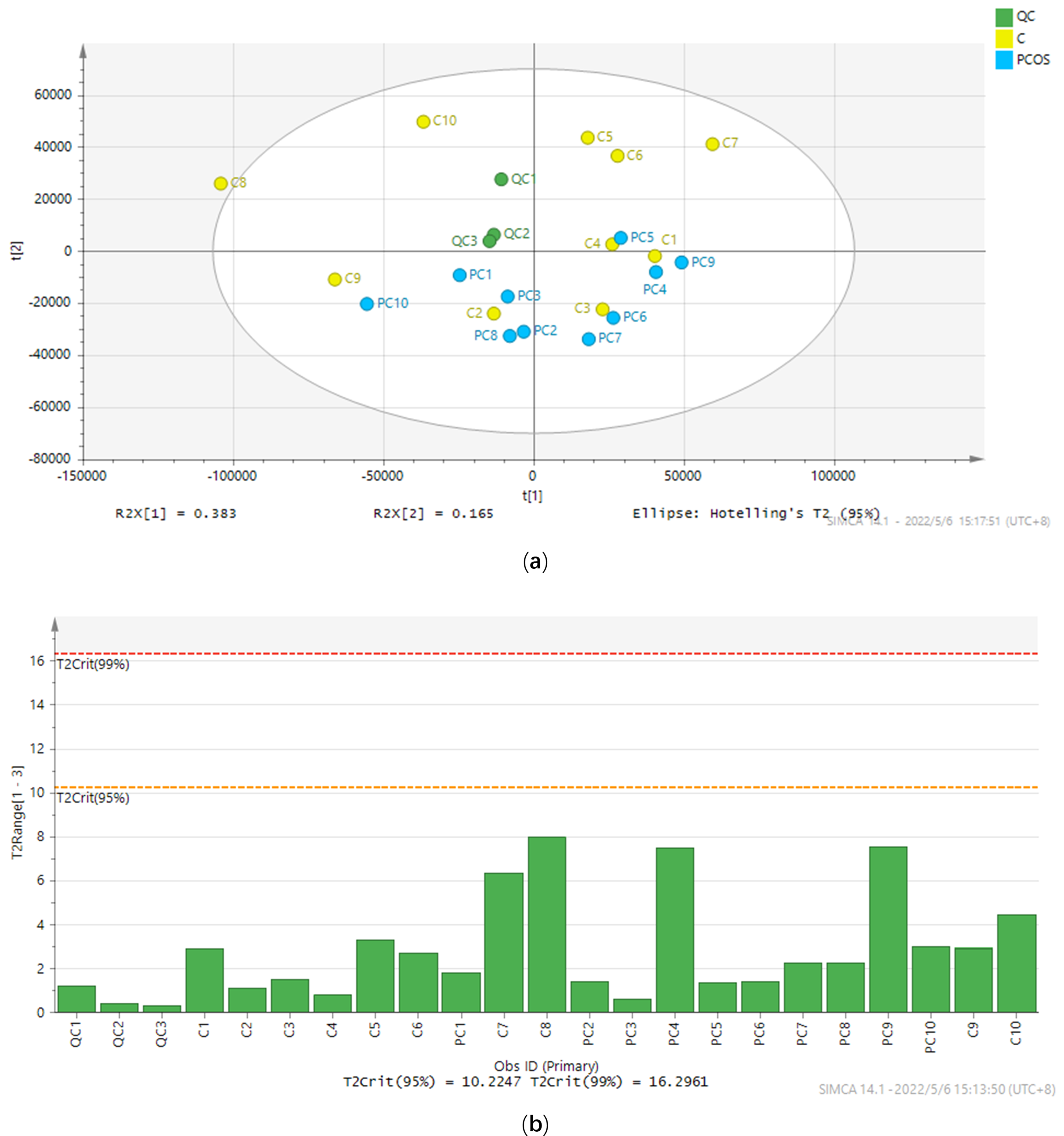

Supplement: Supplementary file 3 — Figure S3. Analysis of follicular fluid negative ion PCA results. [file RMB2-24-e12664-s005.tif]

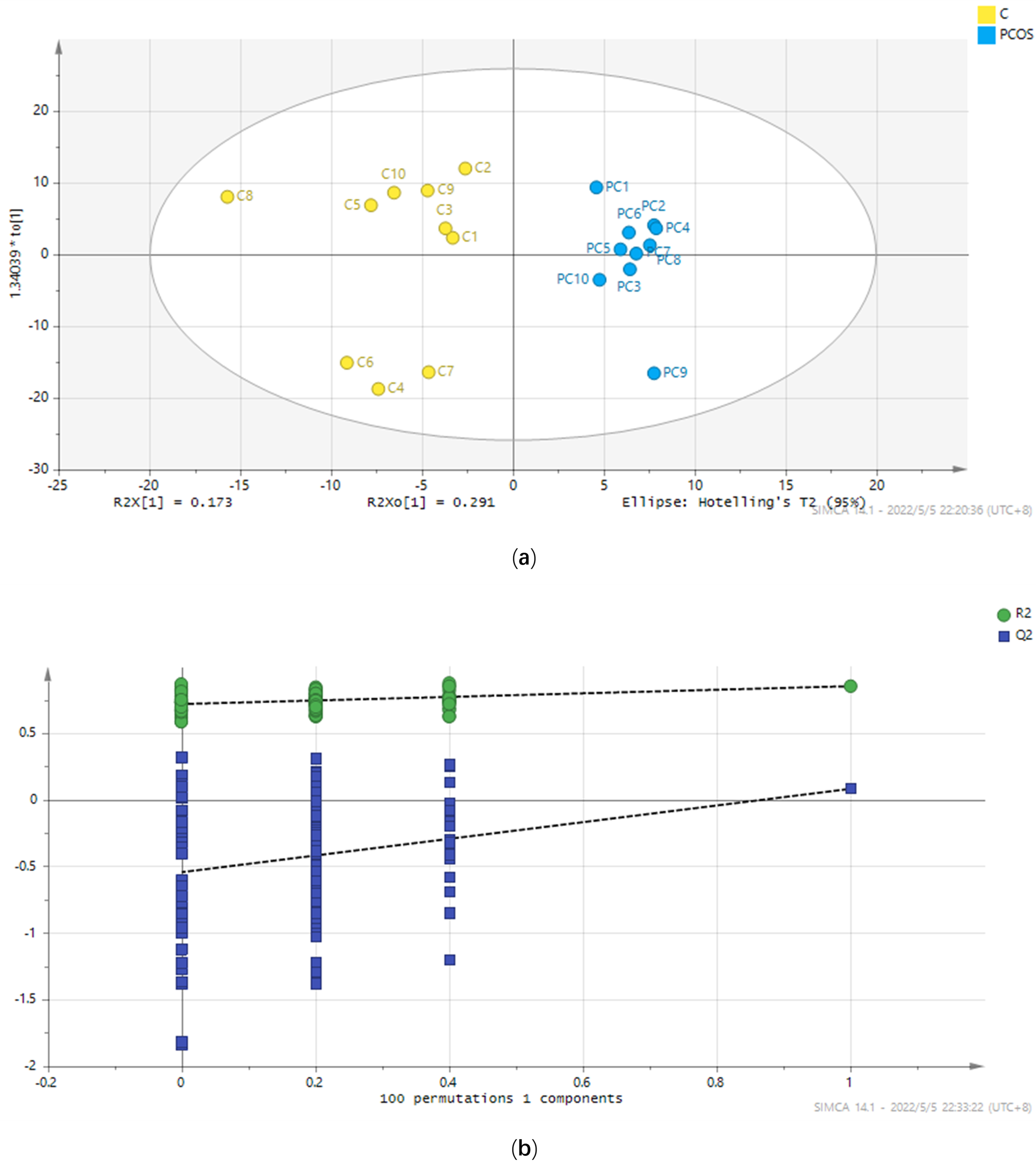

Supplement: Supplementary file 4 — Figure S4. Results of multivariate statistical analysis of metabolomics of follicular fluid positive ions. [file RMB2-24-e12664-s008.tif]

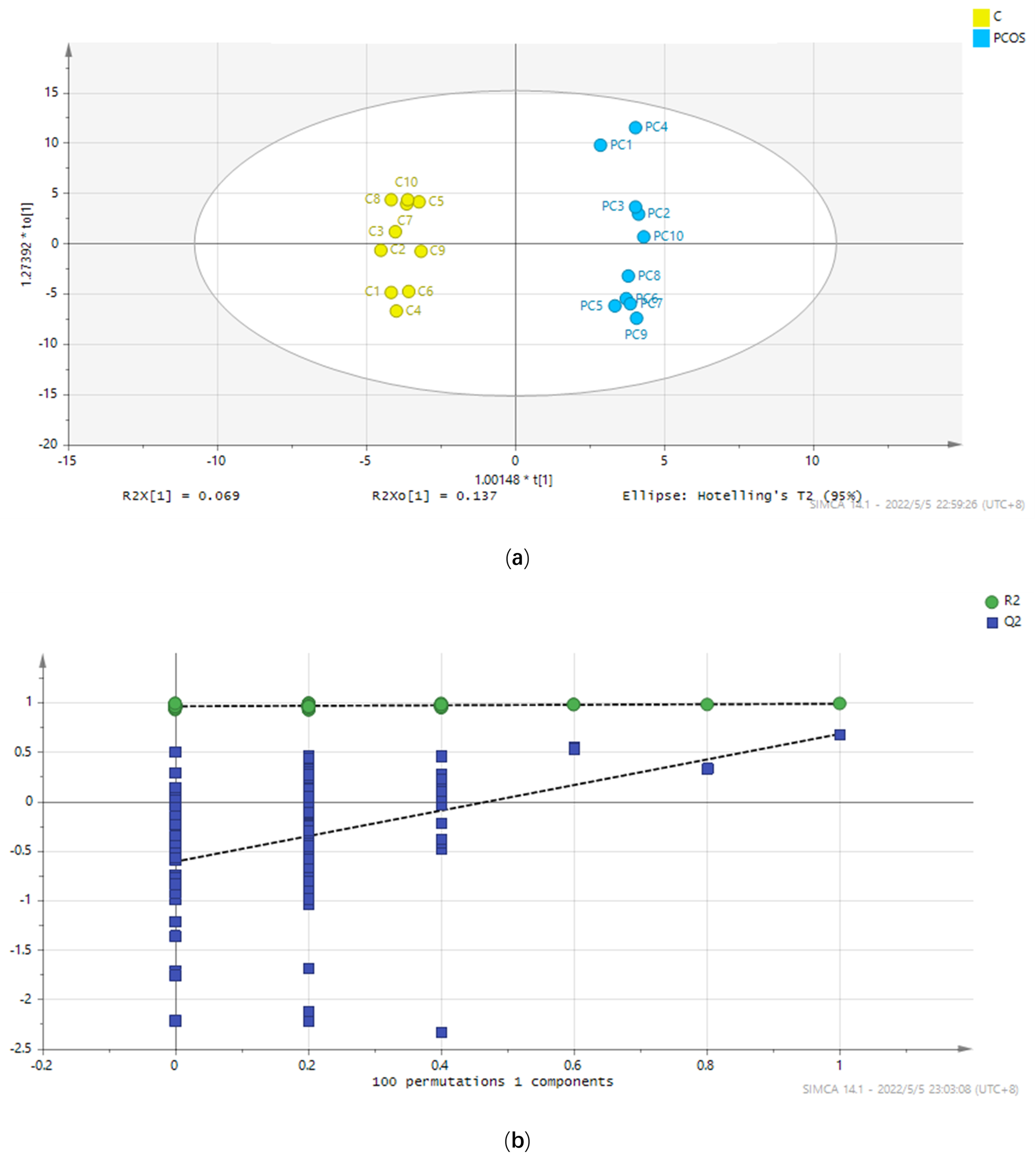

Supplement: Supplementary file 5 — Figure S5. Results of multivariate statistical analysis of metabolomics of follicular fluid negative ions. [file RMB2-24-e12664-s009.tif]
